# Glycome analysis of extracellular vesicles derived from human induced pluripotent stem cells using lectin microarray

**DOI:** 10.1038/s41598-018-22450-2

**Published:** 2018-03-05

**Authors:** Sayoko Saito, Keiko Hiemori, Kayo Kiyoi, Hiroaki Tateno

**Affiliations:** Biotechnology Research Institute for Drug Discovery (BRD), National Institute of Advanced Industrial Science and Technology (AIST), Tsukuba Central 2, 1-1-1 Umezono, Tsukuba, Ibaraki 305-8568 Japan

## Abstract

Glycans are one of the major building blocks of extracellular vesicles (EVs). However, their roles and applications have not been completely explored. Here, we analyzed the glycome of EVs derived from human induced pluripotent stem cells (hiPSCs) using high-density lectin microarray. The glycan profiles of hiPSC-derived EVs were different from those of non-hiPSC-derived EVs. Moreover, rBC2LCN that shows specific binding to hiPSCs, showed strong specificity for hiPSC-derived EVs but not non-hiPSCs-derived EVs. Further, other hiPSC-specific probes, such as anti-TRA-1-60, anti-SSEA4, and anti-R-10G, exhibited specific, but weaker binding to hiPSC-derived EVs than rBC2LCN. We then developed a sandwich assay using rBC2LCN and a phosphatidylserine receptor, Tim4, to specifically detect hiPSC-derived EVs. The Tim4–rBC2LCN sandwich assay allowed for specific detection of hiPSC-derived EVs but not non-hiPSC-derived EVs, indicating that rBC2LCN could also be used for the specific detection of hiPSC-derived EVs. Together, our findings demonstrate that the characteristic glycan signature of hiPSCs are retained by EVs derived from them. The EV glycome could be novel targets for the identification and characterization of stem cells for use in regenerative medicine.

## Introduction

Extracellular vesicles (EVs) are nano- and micro-sized membrane vesicles that comprise a lipid bilayer membrane, including exosomes of endocytic origin, microvesicles generated plasma membrane budding, and apoptotic bodies^1^. EVs have emerged as important mediators of intercellular communication because they function as vehicles for proteins, lipids, and nucleic acids during the transmission of biological signals between cells^2^. EVs and their components are recognized as a novel class of therapeutic targets because these nanoparticles are tightly linked to tumorigenesis and the spread of viruses and prion proteins^2^. EVs are also exploited as biomarkers of various diseases as well as potential therapeutic agents for tissue regeneration and immune response modulation^2^.

Glycosylation is a major post-translational modification in which glycan molecules are attached to many secreted and membrane proteins and lipids. Glycans are bound to the surface of cells as a dense array and play key roles in immunity, development, neural functions, and differentiation^3^. Cell surface glycan structures are dramatically altered during development, cellular activation, differentiation, and malignant transformation, and are often referred to as the “cell signatures” that closely reflect cellular backgrounds and conditions^4^. Indeed, cell surface molecules, such as stage-specific embryonic antigens (SSEA-3/4)^5^, tumor-rejection antigens (TRA-1-60, TRA-1-81)^6–8^, and R-10G^9^, are glyco-biomarkers used to identify human pluripotent stem cells (hPSCs), including human induced pluripotent stem cells (hiPSCs) and human embryonic stem cells (hESCs).

Previously, we performed comprehensive glycome analysis of a large number of hPSCs (114 types of hiPSCs and nine types of hESCs) using high-density lectin microarray, DNA microarray, and high-performance liquid chromatography (HPLC) combined with mass spectrometry (MS)^4,10,11^. We demonstrated that three glycan epitopes, including α2-6Sia, α1-2Fuc, and type1 *N*-acetyllactosamine (LacNAc), are up-regulated in pluripotent state^4,10,11^. Furthermore, we showed that the recombinant N-terminal domain of the lectin BC2L-C derived from *Burkholderia cenocepacia* (rBC2LCN) specifically binds to hPSCs but not non-hPSCs^4^. The recombinant lectin, rBC2LCN, is used to stain fixed cells as well as live cells by adding it to the cell culture media^12^. This staining is specific to undifferentiated cells and rapidly diminishes upon their differentiation^12^. The analysis of the binding mechanism of rBC2LCN to hPSCs revealed that rBC2LCN recognizes Fucα1-2 Galβ1-3GalNAc displayed on podocalyxin, a hyperglycosylated sialomucin^4,13,14^. rBC2LCN lectin has been applied to detect and eliminate hPSCs residing in hiPSC-derived cell therapy products to overcome the tumorigenicity of hPSCs, which is a major concern in regenerative medicine^15,16^.

In addition to proteins, nucleic acids, and lipids, EVs are also composed of glycans. EV glycome may provide vital clues for a better understanding the biogenesis, release, and transfer of vesicles^17^. However, little is known regarding glycans on EVs. Do glycans on EVs change depending on cell types and cellular conditions? More specifically, do hiPSCs-derived EVs carry hiPSC glycan markers? Such basic questions remain unclear.

Here, we performed glycome analysis of EVs derived from hiPSCs compared with those derived from non-hiPSCs using high-density lectin microarray^4^. Unsupervised hierarchical cluster analysis of the microarray data revealed that hiPSC-derived EVs were clearly distinct from non-hiPSC-derived EVs. Detailed analysis of the results obtained by lectin microarray and flow cytometry revealed that hiPSC-derived EVs carry characteristic features of cell surface glycans. rBC2LCN, a specific lectin for hPSCs, bound to hiPSC-derived EVs, but not to non-hiPSC-derived EVs. One of the glycoprotein ligands of rBC2LCN on EVs was identified as podocalyxin, which is a cell surface glycoprotein ligand of rBC2LCN. Other hiPSC surface glycan markers were also detected on the surface of EVs. Finally, we developed a sandwich assay to specifically detect hiPSC-derived EVs using rBC2LCN and Tim4, which binds to phosphatidylserine (PS). rBC2LCN is useful for the specific detection of hiPSC-derived EVs. The EV glycome reflects its cellular origin, which could be a novel target for the development of the quality control system of stem cells used for regenerative medicine.

## Results

### Glycan profiling of hiPSC-derived EVs using high-density lectin microarray

**Figure 1:**
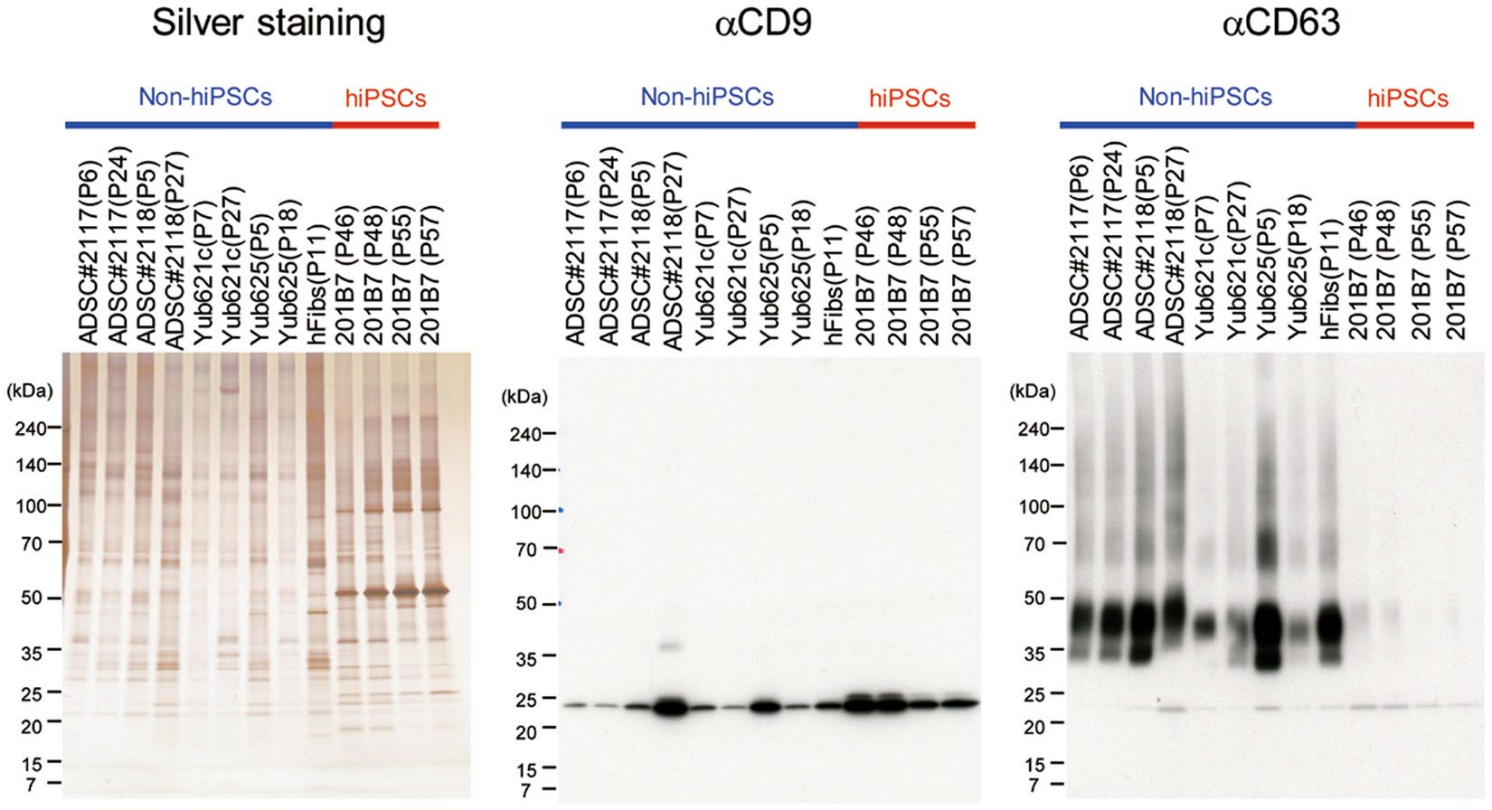
EVs were purified from hiPSCs and non-hiPSCs (Table S1) by Tim4-immobilized beads. Purified EVs (0.2 μg) were electrophoresed under non-reducing conditions on 5–20% polyacrylamide gels and visualized with sliver staining. Separated proteins were transferred to a PVDF membrane and blotted with anti-CD9 and anti-CD63.

EVs were captured from the cell culture media of the 201B7 hiPSCs, human adipose-derived mesenchymal stem cells (ADSC#2117, #2118), human chondrocytes (Yub621c, Yub625), and human dermal fibroblasts (hFibs) (Table S1) by beads immobilized with mouse Tim4, which specifically binds to PS on EVs in a Ca^2+^-dependent manner^18^. The purified EVs were separated using sodium dodecyl sulfate (SDS) polyacrylamide gel electrophoresis (PAGE) and detected by silver staining (Fig. 1). The protein bands were transferred to polyvinylidene difluoride (PVDF) membrane and immunoblotted with antibodies against exosome markers, such as CD9 and CD63 (Fig. 1). A major band was detected at 24 kDa with anti-CD9 antibody for all of the EVs tested. Immunoblotting with anti-CD63 antibody exhibited strong and diffuse protein bands in the size range of 35–50 kDa for non-hiPSC-derived EVs, but only weakly stained bands for hiPSC-derived EVs, indicating that EVs expressed CD9 and CD63 to varying degrees. EVs purified from 201B7 hiPSCs, ADSCs, and hFibs exhibited an average size of 179, 211, and 163 nm, respectively (Fig. S1), indicating that the prepared EV fractions were devoid of large EVs, such as apoptotic cells.

**Figure 2:**
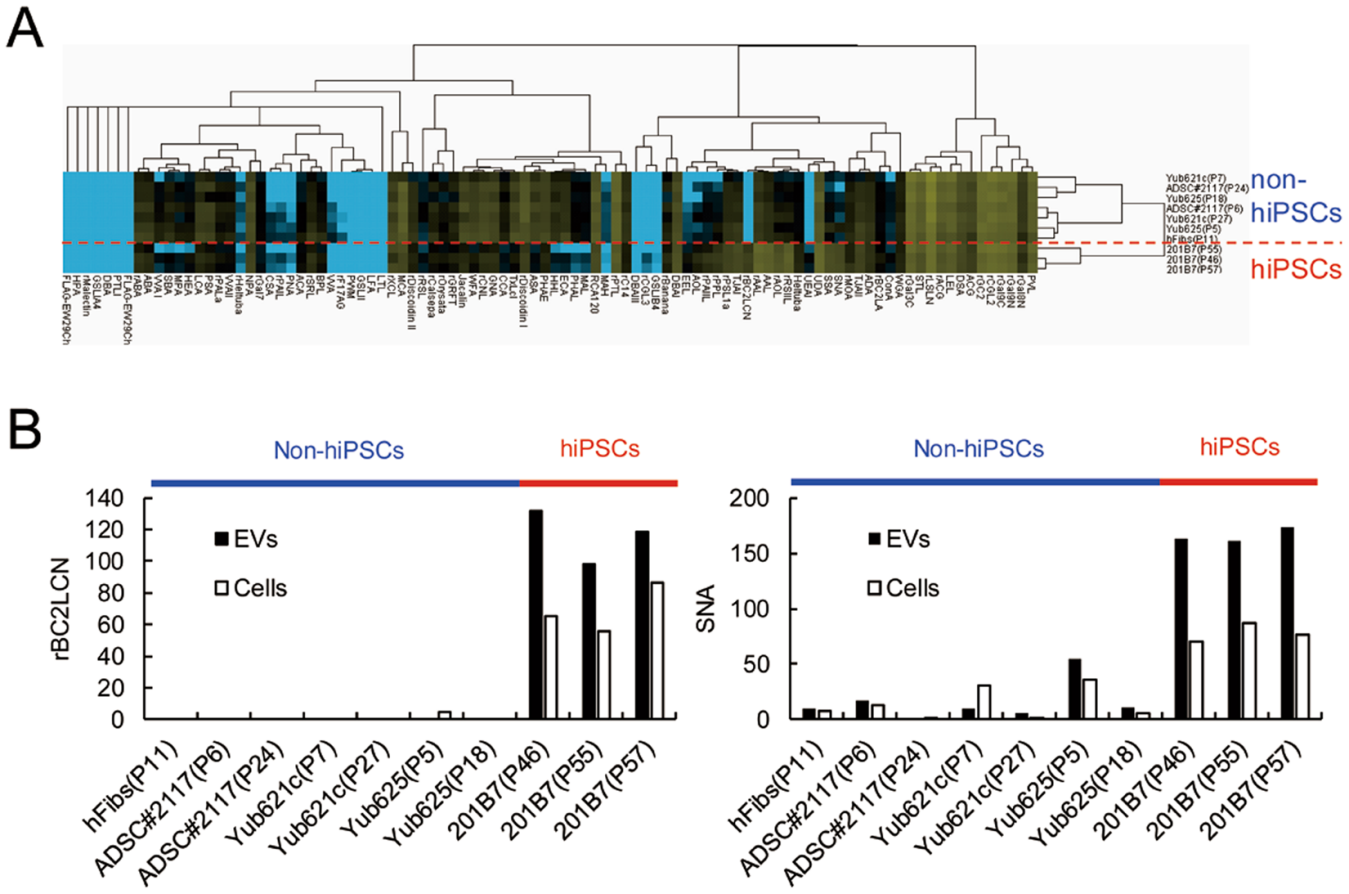
Purified EVs were analyzed by high-density lectin microarray containing 96 lectins. (**A**) Unsupervised cluster analysis of fluorescently labeled EV signals. Yellow: high signal, black: moderate signal, blue: low signal. (**B**) Bar graphs showing rBC2LCN (left) and SNA (right) signals against cell hydrophobic fractions and EVs. Data are shown as the mean of triplicate spots.

The purified EVs were fluorescently labeled with Cy3-*N*-hydroxysuccinimide ester and incubated with high-density lectin microarray containing 96 lectins (Table S2). Fluorescence signals were normalized by the average signals of 96 lectins and analyzed by unsupervised hierarchical cluster analysis. As shown in Fig. 2A, EVs derived from hiPSCs and non-hiPSCs formed two distinct clusters, indicating that the glycan profiles of hiPSC-derived EVs were highly different from those of non-hiPSC-derived EVs, which is consistent with the results of the cell surface glycome^4,12^.

A comprehensive cell surface glycome profiling of hiPSCs revealed that rBC2LCN lectin binds specifically to hPSCs, but not to non-hPSCs^4^. In addition, α2-6Sia-binding lectins showed stronger binding to hiPSCs than non-hiPSCs^4^. The recombinant lectin, rBC2LCN exhibited specific binding to hiPSC-derived EVs, but not to non-hiPSC-derived EVs (Fig. 2B, left panel). Additionally, α2-6Sia-binding lectins, such as SNA, showed stronger binding to hiPSC-derived EVs than to non-hiPSC-derived EVs (Fig. 2B, right panel). These results demonstrate that hiPSC-derived EVs retain the characteristic features of glycan structures expressed on the surface of hiPSCs.

### Flow cytometry analysis of EVs

**Figure 3:**
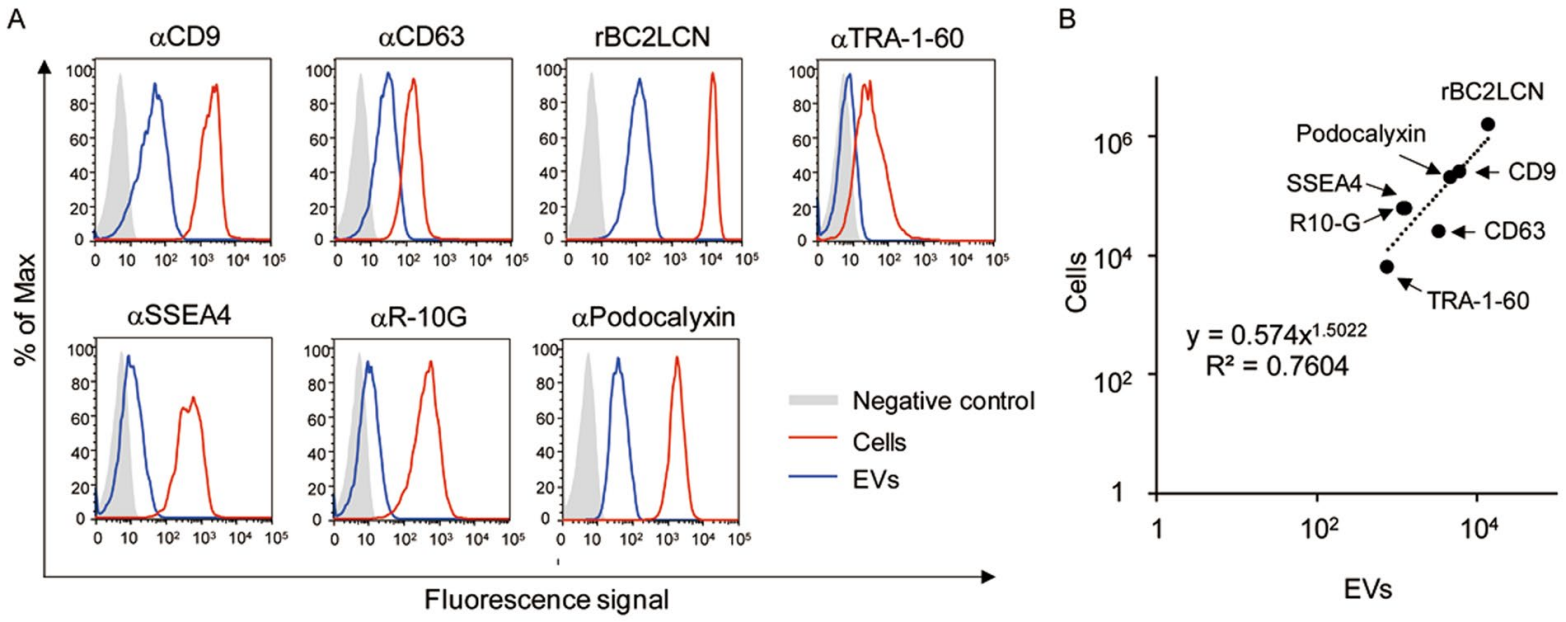
Flow cytometry analysis. (**A**) Expression of exosome markers (CD9 and CD63) and hiPSC markers [rBC2LCN, TRA-1-60, SSEA4, R-10G, podocalyxin (Pod)] on EVs and cells. (**B**) Correlation between marker expression of cells (y-axis) and EVs (x-axis).

Next, we analyzed the expression of hiPSC markers including rBC2LCN ligands, SSEA4, and TRA-1-60 on EVs by flow cytometry. Cell culture supernatants of 201B7 hiPSCs were incubated with Tim4-immobilized beads and captured EVs were detected with fluorescently labeled antibodies or lectins. As shown in Fig. 3A, EVs captured with Tim4-immobilized beads could be detected with exosome marker antibodies, anti-CD9 and anti-CD63. In consistent with the results obtained by western blotting (Fig. 1), anti-CD9 exhibited strong binding to hiPSC-derived EVs, whereas anti-CD63 showed weaker binding (Fig. 3A). Strong binding of rBC2LCN to hiPSCs as well as hiPSC-derived EVs was observed by flow cytometry (Fig. 3A). Other hiPSC marker antibodies, such as anti-TRA-1-60, anti-SSEA4, and anti-R-10G, bound to EVs as well as cells; however, their fluorescence signal intensities were significantly weaker than that of rBC2LCN. hiPSC marker expression on EVs showed a positive correlation with cell surface expression (R^2^ = 0.76), suggesting that the glycans on EVs reflect cell surface glycan expression (Fig. 3B).

### Podocalyxin is a glycoprotein ligand of rBC2LCN on hiPSC-derived EVs

**Figure 4:**
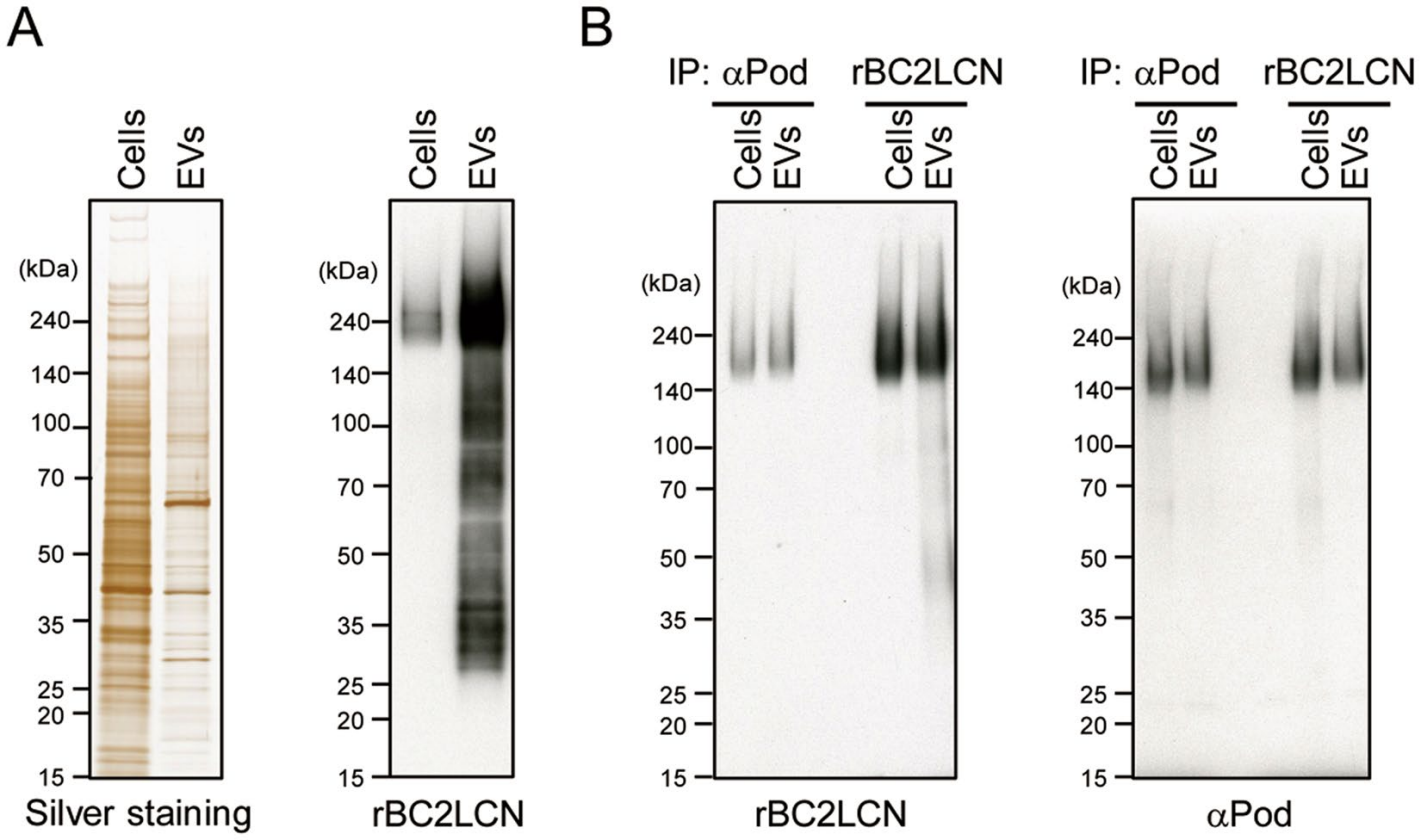
Podocalyxin is a glycoprotein ligand of rBC2LCN. (**A**) Hydrophilic fractions (labeled as “*cells*”, 0.4 μg) and EVs (0.4 μg) of 201B7 hiPSCs were separated by SDS PAGE and detected by silver staining. Separated proteins were transferred to PVDF membranes and blotted with HRP-labeled rBC2LCN. (**B**) Hydrophilic fractions (labeled as “*cells”*, 4 μg) and EVs of 201B7 hiPSCs (4 μg) were captured with anti-podocalyxin (αPod) or rBC2LCN and blotted with HRP-labeled rBC2LCN or HRP-labeled anti-podocalyxin.

Because rBC2LCN exhibited specific binding to hiPSC-derived EVs, we searched for glycoprotein ligands of rBC2LCN in hiPSC-derived EVs. Hydrophilic fractions and EVs prepared from hiPSCs were separated by SDSPAGE and hybridized with horseradish peroxidase (HRP)-conjugated rBC2LCN. As shown in Fig. 4A, strong and diffuse protein bands were detected at approximately 240 kDa in hydrophilic fractions prepared from hiPSCs. A strong band of similar molecular weight (240 kDa) was also detected in EVs. In our previous report, the high-molecular weight protein band stained with rBC2LCN in hydrophilic fractions of hPSCs was identified as podocalyxin^19^. Therefore, we postulated that the high-molecular weight band stained with rBC2LCN in hiPSC-derived EVs might also be podocalyxin. As shown in Fig. 4B, left panel, rBC2LCN bound to podocalyxin immunoprecipitated from EVs as well as cell hydrophilic fractions. Furthermore, glycoproteins immunoprecipitated with rBC2LCN-immobilized magnetic beads showed diffuse protein bands approximately 240 kDa when hybridized with anti-podocalyxin antibody (pAb) (Fig. 4B, right panel). These results suggest that podocalyxin is a glycoprotein ligand of rBC2LCN on EVs.

### Development of a sandwich assay to detect hiPSC-derived EVs

**Figure 5:**
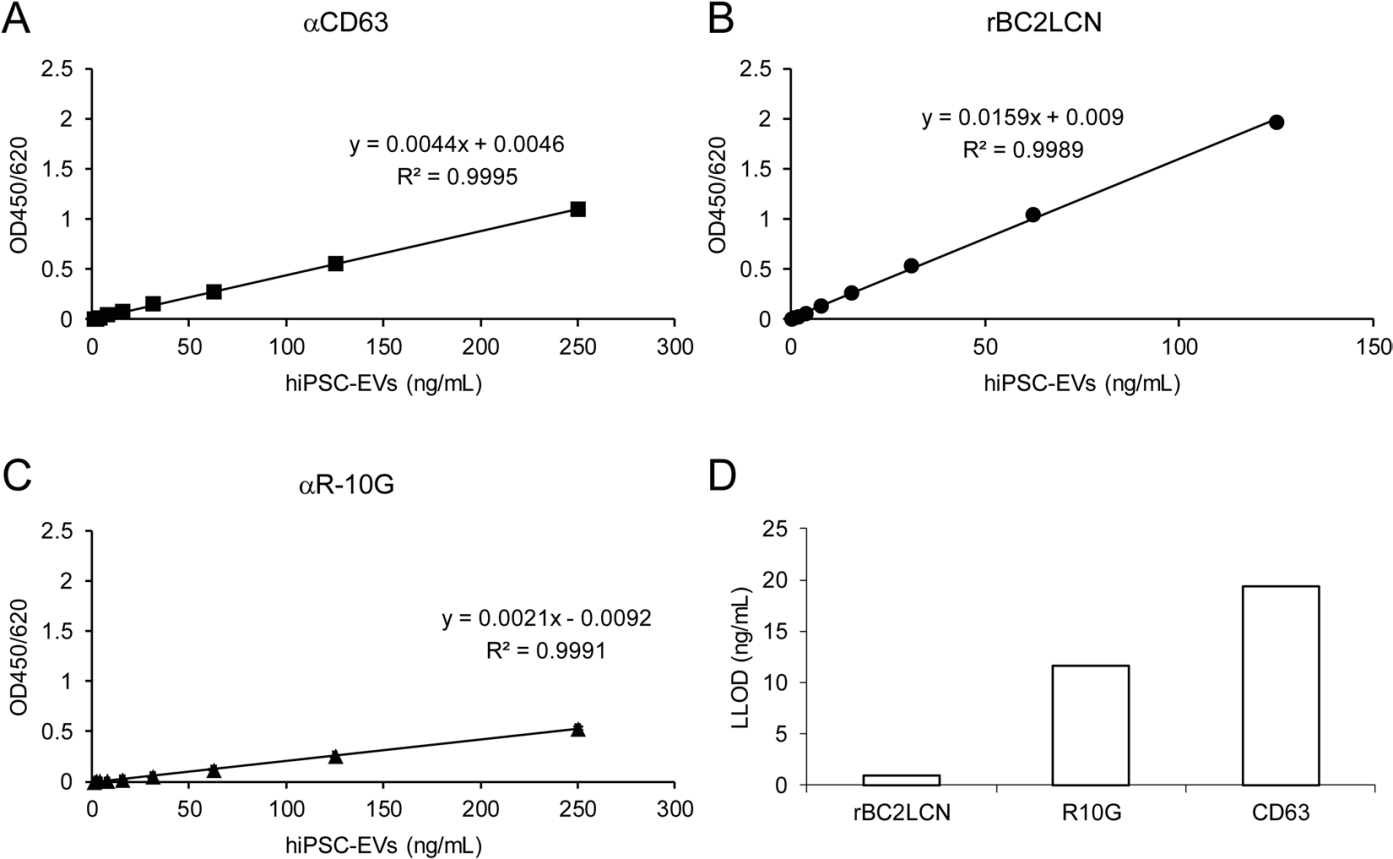
Detection of hiPSC-derived EVs by a sandwich assay. Tim4-coated microtiter plates were incubated with varying concentrations of purified hiPSC-derived EVs and detected with HRP-labeled anti-CD63 (**A**), rBC2LCN (**B**), and anti-R-10G (**C**). Absorbance was measured at OD450/650. Data shown represent the mean ± SD of triplicate samples. (**D**) The Lower limit of detection (LLOD) was calculated as the mean plus 3.3-fold the standard deviation of the measurement of the negative control media. Data shown represent the mean ± SD of triplicate samples.

Because hiPSC markers, such as rBC2LCN ligands, are expressed on hiPSC-derived EVs, we investigated whether rBC2LCN can be used to detect hiPSC-derived EVs. Microtiter plates immobilized with Tim4 were incubated with varying concentrations of hiPSC-derived EVs and overlaid with HRP-labeled anti-CD63, rBC2LCN, and anti-R-10G antibodies. As shown in Fig. 5, standard curves with R^2^ > 0.99 were obtained with all probes. rBC2LCN produced stronger signals than anti-CD63 and anti-R-10G (Fig. 5), which was in agreement with the results obtained by flow cytometry (Fig. 3). The lower limit of detection (LLOD) of hiPSC-derived EVs using rBC2LCN was 0.97 ng/mL, which was lower than that with R-10G (11.7 ng/mL) and anti-CD63 (19.4 ng/mL), indicating that the Tim4-rBC2LCN sandwich assay was the most sensitive system to detect hiPSC-derived EVs (Fig. 5D).

### Specific detection of hiPSC-derived EVs in cell culture media with Tim4-rBC2LCN sandwich assay

**Figure 6:**
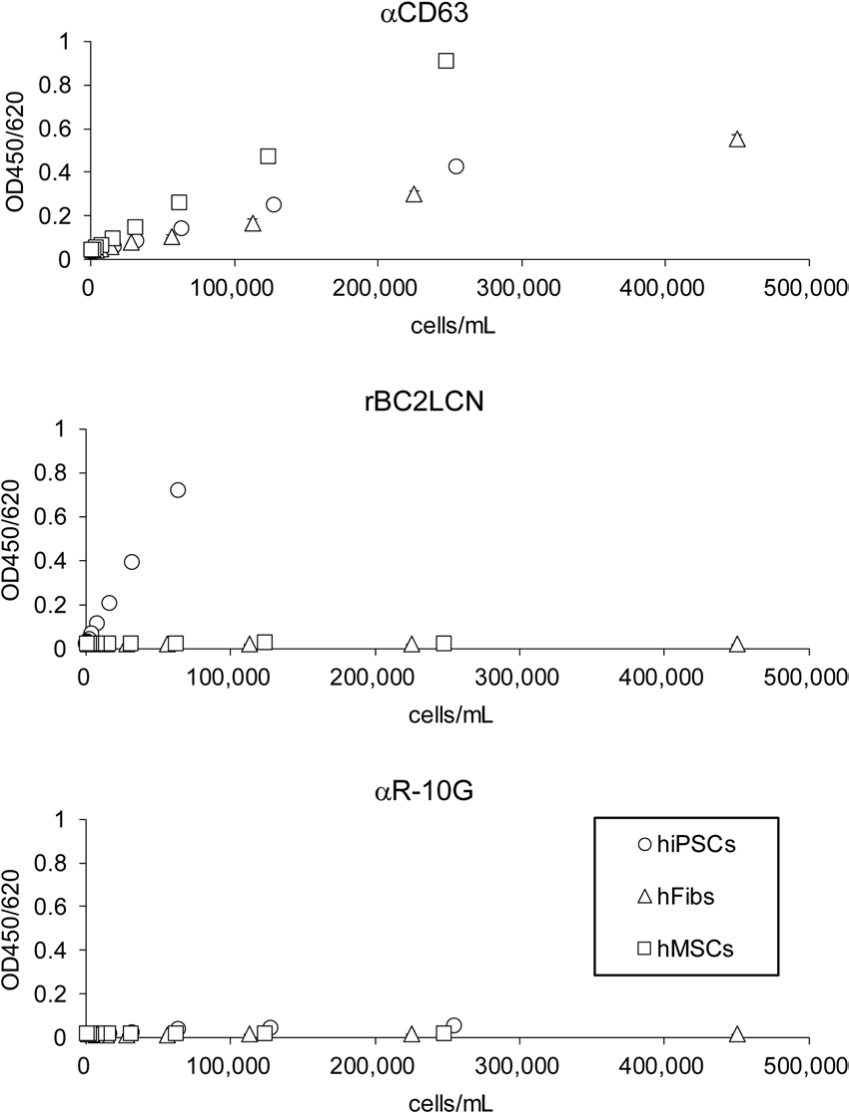
Cell culture supernatants of 201B7 hiPSCs (hiPSCs, *circle*), hFibs, and human bone marrow-derived mesenchymal stem cells (hMSCs) were serially diluted with PBS and reacted with Tim4-coated microtiter plate. Captured Tim4-positive EVs were detected with HRP-labeled anti-CD63 (top), HRP-rBC2LCN (middle), and HRP-R-10G (bottom). Absorbance was measured at OD450/650. Data shown represent the mean ± SD of triplicate samples.

We assessed the ability of the sandwich assay to specifically measure hiPSC-derived EVs. Microtiter plates immobilized with Tim4 were incubated with the cell culture supernatants derived from 201B7 hiPSCs, hFibs, and human bone marrow-derived mesenchymal stem cells (hMSCs), and immunodetected with HRP-labeled anti-CD63, rBC2LCN, and anti-R-10G antibodies. In addition to hiPSCs, hFibs and hMSCs were detected with the Tim4-anti-CD63 sandwich assay (Fig. 6). By contrast, only hiPSCs were detected with the Tim4-rBC2LCN sandwich assay (Fig. 6). Low signals were obtained with the Tim4-anti-R-10G sandwich assay, presumably due to the weak binding of anti-R-10G to hiPSC-derived EVs by flow cytometry (Fig. 3). These results demonstrated that the Tim4-rBC2LCN sandwich assay was a highly specific and sensitive method for the detection of hiPSC-derived EVs.

### Detection of hiPSC-derived EVs during endodermal differentiation

We applied the Tim4-rBC2LCN sandwich assay for the detection of hiPSC-derived EVs during endodermal differentiation. The 201B7 hiPSCs differentiated into endoderm for 7 days were analyzed for the expression of cell surface markers using flow cytometry. While the expression of endoderm markers, Sox17 and FoxA2 was not detected at day 0, it gradually increased during endoderm differentiation (Fig. S2). By contrast, the binding of rBC2LCN gradually decreased during differentiation (Fig. S2). Together, these data provided evidence for the differentiation of hiPSCs into endoderm.

**Figure 7:**
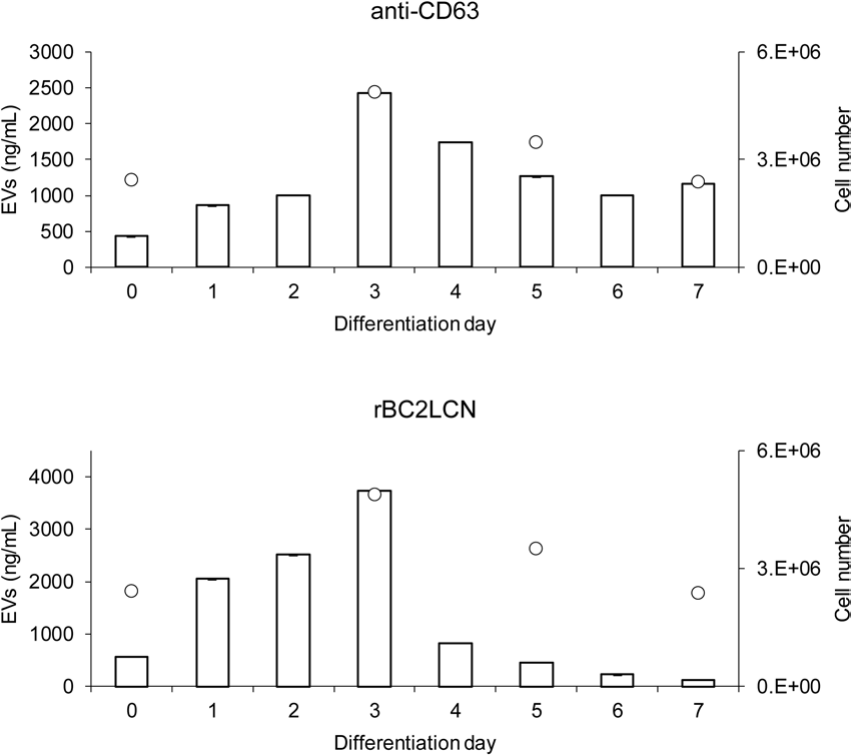
Detection of EVs during endoderm differentiation. The 201B7 hiPSCs were differentiated into endoderm for 7 days. Cell culture supernatants were reacted with Tim4-coated microtiter plate. Captured Tim4-positive EVs were detected with HRP-labeled anti-CD63 (top) and HRP-rBC2LCN (bottom). Absorbance was measured at OD450/650. Data shown represent the mean ± SD of triplicate samples. Open circles denote cell number.

The amount of EVs during endoderm differentiation was then measured with sandwich assays. Cell culture supernatants were incubated with Tim4-coated microplates and detected with HRP-labeled anti-CD63 or rBC2LCN. As shown in Fig. 7, the amount of the total EVs detected by the Tim4-anti-CD63 sandwich assay was directly proportional to the number of cells, regardless of the state of differentiation. EVs secreted from differentiated cells day 7 were also detected with the Tim4-anti-CD63 sandwich assay. The Tim4-rBC2LCN sandwich assay showed the highest value at day 3 of differentiation and dramatically decreased at day 4. These data showed the Tim4-rBC2LCN sandwich assay specifically detected the hiPSC-derived EVs.

## Discussion

To understand the cell surface glycome of hiPSCs and hESCs in a comprehensive way, we previously performed the glycome analysis of a large set of hiPSCs and hESCs using high-density lectin microarray^4^. In unsupervised hierarchical clustering, undifferentiated hPSCs and differentiated non-hPSCs were clearly separated into two different clusters, indicating that hPSCs show different cell surface glycome from non-hPSCs. The expression of three glycan epitopes, including α2-6Sia, α1-2Fuc, and type1 LacNAc, was strongly indicated to be increased upon the induction of pluripotency^4^. Consistent with these findings, responsible glycosyltransferase genes, such as *ST6Gal1/2*, *FUT1/2*, and *B3GalT5*, involved in the synthesis of these glycan epitopes showed significant increases in expression levels^4^. In addition, we found that rBC2LCN bound all the undifferentiated hPSCs tested but not differentiated non-hPSCs^4,14^. In this study, the glycome of hiPSC-derived EVs was analyzed by applying the same strategy using high-density lectin microarray. Although several groups reported that the glycan profiles of EVs were distinct from the surface profiles of the derived cells^20,21^, the glycome of hiPSC-derived EVs was found to retain the characteristic features of cell surface glycome of hPSCs as described below:

1. hiPSC-derived EVs exhibited clearly different glycan profiles from those of non-hiPSC-derived EVs
2. rBC2LCN showed specific binding to hiPSC-derived EVs but no or little binding to non-hiPSC-derived EVs
3. α2-6Sia-binding lectin (SNA) showed higher signal for undifferentiated hiPSCs than for differentiated non-hiPSCs.

These data were not the result of the contamination of live cells in the prepared EV fractions because live cells and large EVs, such as apoptotic bodies, were completely removed from cell culture supernatants by three continuous centrifugation and Tim4-affinity purification. Consistently, negligible amounts of large particles with >500 nm size were detected in the prepared EV fractions. The average diameter of EVs prepared from ADSC was 225 nm, which was similar to the previous report (178 nm)^21^.

hiPSC markers, such as SSEA4, TRA-1-60, and rBC2LCN ligands, were also detected on EVs by flow cytometry. The expression of hiPSC markers on EVs showed a strong correlation with that of cell surface expression. Together, these data suggest that the glycan signature of hiPSC-derived EVs is similar to that of cell surface and can be used for the quality control of stem cells used for regenerative medicine. However, since some membrane proteins such as tetraspanins are enriched in EVs^22^, it is possible that some glycoconjugates might be specifically enriched in EVs. Further detailed analysis is essential to clarify the structure of the EV glycome and its relationship with the cell surface glycome.

rBC2LCN showed strong binding to hiPSC-derived EVs as well as hiPSCs. Taking advantage of this finding, we developed a sandwich assay to specifically detect hiPSC-derived EVs. While the Tim4-anti-CD63 sandwich assay detected EVs regardless of the cell type, the Tim4-rBC2LCN sandwich assay detected hiPSC-derived EVs and not those derived from hMSCs and hFibs. Tim4-rBC2LCN sandwich assay showed lower LLOD values to detect hiPSC-derived EVs (LLOD = 0.97 ng/mL) than Tim4-αCD63 (LLOD = 19.4 ng/mL) and Tim4-R-10G (LLOD = 11.7 ng/mL), suggesting that the Tim4-rBC2LCN sandwich assay was determined as the most sensitive system to detect hiPSC-derived EVs. Moreover, the Tim4-rBC2LCN sandwich assay was capable of monitoring the concentration of hiPSC-derived EVs during endodermal differentiation. It should be noted, however, different clones of antibodies or different analytical condition might provide different sensitivity in sandwich assay.

Since glycans are located on the surface of EVs, their roles in EV function, biogenesis, release, and transfer are critical^17^. However, the structure and function of EVs are not yet completely understood. In this study, we analyzed the glycome of EVs derived from hiPSCs and compared these with the glycomes of hMSCs, chondrocytes, and hFibs and demonstrated that hiPSC-derived EVs carry characteristic glycome signature of their cellular origin. We also developed a method to specifically detect hiPSC-derived EVs, demonstrating that the EV glycome is useful for the quality control of stem cells used for regenerative medicine.

## Materials and Methods

### Cell culture

Cells used for the preparation of EVs are summarized in Table S1. The 201B7 hiPSCs were cultured in 2.5 mL of serum-free mTeSR1 (VERITAS, Tokyo, Japan) in 6 cm dishes coated with Matrigel (BD, Tokyo, Japan)^23,24^. Cell culture media were recovered from live cells and used for the preparation of EVs. Non-hiPSCs, including hFibs, ADSCs, and human chondrocytes (Yub621c, Yub625) were cultured in 10 mL of serum-free MesenPRO RS™ Basal Medium (Cat#: 12746-012, Thermo Fisher Scientific K.K., Kanagawa, Japan,) supplemented with 2 mM L-glutamine and 1% penicillin-streptomycin on 10 cm cell culture dishes. Cells were counted with a TC20 Automated Cell Counter (Bio-Rad Laboratories, Inc., Tokyo, Japan). Cell culture supernatant was recovered and stored at −80 °C until use.

### Preparation of EVs and cell membrane fractions

The recovered cell culture media (30–40 mL) were centrifuged at 300 × g for 5 min, 1,200 × g for 20 min, and 10,000 × g for 30 min at 4 °C to remove cells, debris, and large EVs, and concentrated to 1 mL by Amicon Ultra-15 Ultracel-100K (Cat#: UFC910024, Merck KGaA, Darmstadt, Germany,) and EVs were prepared using MagCapture™ Exosome Isolation Kit PS (Cat#: 293-77601, Wako Pure Chemical Industries, Ltd., Osaka, Japan,) according to the manufacturer’s instructions^18^. Particle sizes of the prepared EVs were analyzed using Nanosight LM10 system (Marvern Instruments Ltd., Worcestershire, UK) equipped with a blue laser. Protein concentration was quantified with the BCA assay (Thermo Fisher Scientific). Cell membrane fractions were prepared using the CelLytic MEM Protein Extraction Kit (Merck KGaA) in accordance with the manufacturer’s instructions.

### SDS PAGE and Western blotting

EVs (0.2 μg) were electrophoresed under non-reducing conditions on 5–20% polyacrylamide gels (Cat#: NTH-676HP, DRC, Tokyo, Japan) and visualized using the Sliver Staining MS kit (Cat#: 293-77601, Wako Pure Chemical Industries, Ltd.) according to the manufacturer’s instructions. The separated proteins were transferred to a PVDF membrane. After blocking with BlockAce (Cat#: BUF029, Funakoshi Co., Ltd., Tokyo, Japan), the membrane was incubated with 500-fold dilution of anti-CD9 (Clone#: Ts9, Cat#: 10626D, Thermo Fisher Scientific K.K.) and 2000-fold dilution of anti-CD63 (Clone#: 8A12, Cat#: SHI-EXO-M02, COSMO BIO Co. Ltd., Tokyo, Japan) followed by 10,000-fold dilution of peroxidase-labeled AffiniPure Goat Anti-Mouse IgG (H+L) (Cat#: 115-035-003, Jackson ImmunoResearch Inc., West Grove, PA).

### Production of high-density lectin microarray

High-density lectin microarray immobilizing 96 lectins was produced as previously described^4,25^. Each lectin was spotted at a concentration of 0.5 mg/mL in triplicates. Lectins immobilized on the array are shown in Table S2.

### Lectin microarray analysis of EVs

After adjusting the protein concentration to 2 μg/mL with PBST [10 mM PBS (pH 7.4), 140 mM NaCl, 2.7 mM KCl, 1% Triton X-100], proteins were labeled with Cy3-*N*-hydroxysuccinimide ester (GE Healthcare Ltd., Tokyo, Japan). After dilution with probing buffer [25 mM Tris-HCl (pH 7.5), 140 mM NaCl, 2.7 mM KCl, 1 mM CaCl_2_, 1 mM MnCl_2_, and 1% Triton X-100] to 0.5 μg/mL, Cy3-labeled protein samples were incubated with the high-density lectin microarray at 20 °C overnight. After washing with probing buffer, fluorescence images were captured using a Bio-Rex scan 200 evanescent-field activated fluorescence scanner (Rexxam Co. Ltd., Kagawa, Japan). The lectin signals of triplicate spots were averaged for each protein sample and normalized relative to the mean value of 96 lectins. Unsupervised clustering was performed with the average linkage method using Cluster 3.0 software. The heat map with clustering was generated using Java Treeview.

### Flow cytometry analysis of EVs

The cell culture media of hiPSCs (30–40 mL) were centrifuged at 300 × g for 5 min, 1,200 × g for 20 min, and 10,000 × g for 30 min at 4 °C to remove cells, debris, and large EVs, and concentrated to 1 mL by Amicon Ultra-15 Ultracel-100K (Cat#: UFC910024, Merck KGaA, Darmstadt, Germany). The precleared and concentrated cell culture supernatants were incubated with Tim4-coated beads (Cat#: 293-77601, Wako Pure Chemical Industries) were incubated with the precleared cell culture supernatants of hiPSCs at 4 °C overnight. The beads were then suspended in PBS containing 1% BSA and 1 mM CaCl_2_, and incubated with R-phycoerythrin (PE)-conjugated rBC2LCN or PE-BSA. The beads were also incubated with a 100-fold dilution of anti-CD9 antibody (clone#: 12A12, SHI-EXO-M01, COSMO BIO Co. Ltd.), a 100-fold dilution of anti-CD63 (Clone#: 8A12, Cat#: SHI-EXO-M02, COSMO BIO Co. Ltd.), anti-podocalyxin goat pAb (Cat#: AF1658, R&D Systems, Inc., Minneapolis, MN), a 100-fold dilution of anti-SSEA4 (clone#: MC-813-70, Cat#: MAB4304, Merck KGaA), anti-TRA1-60 (clone#: TRA-1-60, Cat#: MAB4360, Merck KGaA), or R-10G (Clone#: R-10G, Cat#: 011-25811, Wako Pure Chemical Industries, Ltd.) followed by R-phycoerythrin (PE)-conjugated AffiniPure Donkey Anti-Goat IgG (H+L) (Cat#: 705-115-147, Jackson ImmunoResearch Inc.,) or PE-Goat Anti-Mouse IgG (Cat#: 550589, BD). The 201B7 hiPSCs were also incubated with the same lectins or antibodies described above. Flow cytometry data were acquired on a CytoFLEX (Beckman Coulter, Inc., Brea, CA) and analyzed using FlowJo software (FlowJo, LLC., Ashland, OR).

### Sandwich assay

Tim4-coated microplates (Cat#: 79201, Wako Pure Chemical Industries, Ltd.) were incubated with 100 μL/well of cell culture supernatants for 2 h at room temperature with agitation. After washing, 100 μL of HRP-labeled anti-CD63, -rBC2LCN (0.1 μg/mL), -R-10G (0.1 μg/mL) were reacted for 1 h at room temperature with agitation. Finally, TMB solution was added and developed for 30 min at room temperature. The reaction was stopped by adding 100 μL of 1 N HCl and detected dominant absorbance at OD450 and secondary wavelength at OD620.

### Endoderm differentiation

The 201B7 hiPSCs were differentiated into endoderm using STEMdiff™ Definitive Endoderm kit (Cat#: 05110, VERITAS). After 3, 5, and 7 days, cells with or without permeabilization were incubated with PE-labeled anti-Sox17 (Clone#: P7-969, Cat#: 561591, BD), anti-FoxA2 (Clone#: N17-280, Cat#: 561589, BD), and rBC2LCN, analyzed by a CytoFLEX and FlowJo software. Cell culture supernatants were recovered and analyzed with the Tim4-anti-CD63 and Tim4-rBC2LCN sandwich assays as described above.

### Immunoprecipitation and Western blotting

Ten μL of Dynabeads M-280 streptavidin (Thermo Fisher Scientific K.K.,) immobilized with biotinylated rBC2LCN or anti-podocalyxin pAb (R&D Systems, Inc.) were incubated with 4 μg of EVs or cell hydrophobic fractions in PBST(10 mM PBS pH 7.4, 140 mM NaCl, 2.7 mM KCl, 1% triton X-100) at 4 °C overnight with agitation. Subsequently, beads were washed with 200 μL of PBST three times and bound proteins were eluted in 50 μL of TBS containing 0.2% SDS at 95 °C for 10 min. The elution fractions were separated with SDS PAGE and immunolabeled with 200-fold dilution of anti-podocalyxin goat polyclonal antibody (Cat#: AF1658, R&D Systems, Inc.) or peroxidase-labeled rBC2LCN (0.1 μg/mL).

## Electronic supplementary material

Supplementary information
