# Supplementary material for: Glycome analysis of extracellular vesicles derived from human induced pluripotent stem cells using lectin microarray
Source: Sci Rep. 2018 Mar 5;8:3997. doi: 10.1038/s41598-018-22450-2 (PMC5838221; doi:10.1038/s41598-018-22450-2)
Supplement: Supplementary file 1 — Supplementary information [file 41598_2018_22450_MOESM1_ESM.pdf]

Supplementary information

Glycome analysis of extracellular vesicles derived from human induced pluripotent stem cells using lectin microarray

Sayoko Saito, Keiko Hiemori, Kayo Kiyoi, Hiroaki Tateno

Table S1. Cells used for the preparation of EVs

Name	Cell type	Supply	Cat# (Lot#)	Passage
hFibs(P11)	Human fibroblasts	ATCC	PCS-201-012 (58605481)	11
ADSC#2117(P6)	Human adipose-derived messenchymal stem cells	ThermoFisher SCIENTIFIC	R7788110 (2117)	6
ADSC#2117(P24)	Human adipose-derived messenchymal stem cells	ThermoFisher SCIENTIFIC	R7788110 (2117)	24
ADSC#2118(P5)	Human adipose-derived messenchymal stem cells	ThermoFisher SCIENTIFIC	R7788110 (2118)	5
ADSC#2118(P27)	Human adipose-derived messenchymal stem cells	ThermoFisher SCIENTIFIC	R7788110 (2118)	27
Yub621c(P7)	Human chondrocytes from polydactylous fingers	RIKEN BRC	RBRC-HMS0013	7
Yub621c(P27)	Human chondrocytes from polydactylous fingers	RIKEN BRC	RBRC-HMS0013	27
Yub625(P5)	Human chondrocytes from polydactylous fingers	RIKEN BRC	RBRC-HMS0038	5
Yub625(P18)	Human chondrocytes from polydactylous fingers	RIKEN BRC	RBRC-HMS0038	18
201B7(P46)	Human induced pluripotent stem cells	RIKEN BRC	HPS0063	46
201B7(P48)	Human induced pluripotent stem cells	RIKEN BRC	HPS0063	48
201B7(P55)	Human induced pluripotent stem cells	RIKEN BRC	HPS0063	55
201B7(P57)	Human induced pluripotent stem cells	RIKEN BRC	HPS0063	57

SupplementaryTable 2. Lectins used for lectin microarray [1]

	Name	Species	Origin	Source[3]	Rough specificity[2]
1	LFA	*Limax flavus*	Natural	EY Lab.	Sia
2	WGA	*Triticum vulgaris*	Natural	EY Lab.	(GlcNAc)n, polySia
3	PVL	*Psathyrella velutina*	Natural	Wako	Sia, GlcNAc
4	MAL	*Maackia amurensis*	Natural	Seikagaku	α2-3Sia
5	MAH	*Maackia amurensis*	Natural	Vector	α2-3Sia
6	ACG	*Agrocybe cylindracea*	Natural	JOM	α2-3Sia
7	rACG	*Agrocybe cylindracea*	*E.coli*	AIST	α2-3Sia
8	rGal8N	*Homo sapiens*	*E.coli*	AIST	α2-3Sia
9	SNA	*Sambucus nigra*	Natural	Seikagaku	α2-6Sia
10	SSA	*Sambucus sieboldiana*	Natural	Vector	α2-6Sia
11	TJAI	*Trichosanthes japonica*	Natural	Vector	α2-6Sia
12	rPSL1a	*Polyporus squamosus*	*E.coli*	AIST	α2-6Sia
13	PHAL	*Phaseolus vulgaris*	Natural	Seikagaku	GlcNAcβ1-6Man (Tetraantenna)
14	DSA	*Datura stramonium*	Natural	Seikagaku	GlcNAcβ1-6Man (Tetraantenna)
15	TxLcI	*Tulipa gesneriana*	Natural	JOM	Galactosylated N-glycans up to triantenna
16	ECA	*Erythrina cristagalli*	Natural	Seikagaku	βGal
17	RCA120	*Ricinus communis*	Natural	Vector	βGal
18	rGal7	*Homo sapiens*	*E.coli*	AIST	Type1 LacNAc, chondroitin polymer
19	rGal9N	*Homo sapiens*	*E.coli*	AIST	GalNAcα1-4Gal (A), PolyLacNAc
20	rGal9C	*Homo sapiens*	*E.coli*	AIST	PolyLacNAc, Branched LacNAc
21	rC14	*Gallus gallus domestic*	*E.coli*	AIST	Branched LacNAc
22	rDiscoidin II	*Dictyostelium dicodeum*	*E.coli*	AIST	LacNAc, Galβ1-3GalNAc (T), GalNAc (Tn)
23	BPL	*Bauhinia purpurea alba*	Natural	Vector	Galβ1-3GlcNAc(GalNAc), α/βGalNAc
24	rCGL2	*Homo sapiens*	*E.coli*	AIST	GalNAcα1-3Gal (A), PolyLacNAc
25	PHAE	*Phaseolus vulgaris*	Natural	Vector	bisecting GlcNAc
26	GSLII	*Griffonia simplicifoli*	Natural	Vector	GlcNAcβ1-4Man
27	rSRL	*Sclerotium rolfsii*	*E.coli*	AIST	Core1,3, agalacto N-glycan
28	UDA	*Urtica dioica*	Natural	Vector	(GlcNAc)n
29	PWM	*Phytolacca americana*	Natural	Vector	(GlcNAc)n
30	rF17AG	*Escherichia coli*	*E.coli*	AIST	GlcNAc
31	rGRFT	*Griffithia sp.*	*E.coli*	AIST	Man
32	NPA	*Narcissus pseudonarcis*	Natural	Seikagaku	Manα1-3Man
33	ConA	*Canavalia ensiformis*	Natural	Vector	M3, Manα1-2Manα1-3(Manα1-6)Man, GlcNAcβ1-2Manα1-3(Manα1-6)Man
34	GNA	*Galanthus nivalis*	Natural	Vector	Manα1-3Man, Manα1-6Man
35	HHL	*Hippeastrum hybrid*	Natural	Vector	Manα1-3Man, Manα1-6Man
36	ASA	*Allium sativum*	Natural	JOM	Galβ1-4GlcNAcβ1-2Man
37	DBAI	*Dioscorea batatas*	Natural	JOM	High-man
38	CCA	*Castanea crenata*	Natural	JOM	Galactosylated N-glycans up to triantenna
39	Heltuba	*Helianthus t uberosus*	Natural	JOM	Manα1-3Man
40	rHeltuba	*Helianthus tuberosus*	*E.coli*	AIST	Manα1-3Man
41	ADA	*Allomyrina dichtoma*	Natural	JOM	α2-6Sia, Forssman, A, B
42	VVAII	*Vicia villosa*	Natural	JOM	Man, Agalacto
43	rOrysata	*Oryza sativa*	*E.coli*	AIST	Manα1-3Man, Highman, biantenna
44	rPALa	*Phlebodium aureum*	*E.coli*	AIST	Man5, biantenna
45	rBanana	*Musa acuminata*	*E.coli*	AIST	Manα1-2Manα1-3(6)Man
46	rCalsepa	*Calystegia sepium*	*E.coli*	AIST	Biantenna with bisecting GlcNAc
47	rRSL	*Ralstonia solanacearum*	*E.coli*	AIST	αMan, α1-2Fuc (H), α1-3Fuc (Lex), α1-4Fuc (Lea)
48	rBC2LA	*Burkholderia cenocepac*	*E.coli*	AIST	aMan, High-man
49	AOL	*Aspergillus oryzae*	Natural	Vector	α1-2Fuc (H), α1-3Fuc (Lex), α1-3Fuc (Lea)
50	AAL	*Aleuria aurantia*	Natural	Vector	α1-2Fuc (H), α1-3Fuc (Lex), α1-4Fuc (Lea)
51	rAAL	*Aleuria aurantia*	*E.coli*	AIST	α1-2Fuc (H), α1-3Fuc (Lex), α1-3Fuc (Lea)
52	rPAIIL	*Pseudomonas aeruginosa*	*E.coli*	AIST	aMan, a1-2Fuc (H), a1-3Fuc (Lex), a1-4Fuc (Lea)
53	rRSIIL	*Ralstonia solanacearum*	*E.coli*	AIST	α1-2Fuc (H), α1-3Fuc (Lex), α1-3Fuc (Lea)
54	rPTL	*Pholiota terrestris*	*E.coli*	AIST	α1-6Fuc
55	PSA	*Pisum sativum*	Natural	Seikagaku	α1-6Fuc up to biantenna
56	LCA	*Lens culinaris*	Natural	Vector	α1-6Fuc up to biantenna
57	rAOL	*Aspergillus oryzae*	*E.coli*	AIST	α1-2Fuc (H), α1-3Fuc (Lex), α1-3Fuc (Lea)
58	rBC2LCN	*Burkholderia cenocepac*	*E.coli*	AIST	Fucα1-2Galβ1-3GlcNAc (GalNAc)
59	LTL	*Lotus tetragonolobus*	Natural	Seikagaku	Lex, Ley
60	UEAI	*Ulex europaeus*	Natural	Vector	α1-2Fuc
61	TJAII	*Trichosanthes japonica*	Natural	Vector	α1-2Fuc
62	MCA	*Momordica charantia*	Natural	JOM	α1-2Fuc
63	GSLI	*Griffonia simplicifoli*	Natural	Seikagaku	αGalNAc (A, Tn), aGal (B)
64	PTLI	*Psophocarpus tetragono*	Natural	Tokyo Kasei	αGalNAc (A, Tn)
65	GSLIA4	*Griffonia simplicifoli*	Natural	EY Lab.	αGalNAc (A, Tn)
66	rGC2	*Geodia cydonium*	*E.coli*	AIST	α1-2Fuc (H), αGalNAc (A), αGal (B)
67	GSLIB4	*Griffonia simplicifoli*	Natural	Vector	αGal (B)
68	rMOA	*Marasmius oreades*	*E.coli*	AIST	αGal (B)
69	EEL	*Euonymus europaeus*	Natural	Vector	αGal (B)
70	rPAIL	*Pseudomonas aeruginosa*	*E.coli*	AIST	α,βGal, αGalNAc (Tn)
71	LEL	*Lycopersicon esculentu*	Natural	Vector	Polylactosamine, (GlcNAc)n
72	STL	*Solanum tuberosum*	Natural	Seikagaku	Polylactosamine, (GlcNAc)n

73	rGal3C	*Homo sapiens*	*E.coli*	AIST	LacNAc, polylactosamine
74	rLSLN	*Laetiporus sulphureus*	*E.coli*	AIST	LacNAc, polylactosamine
75	rCGL3	*Coprinopsis cinerea*	*E.coli*	AIST	LacDiNAc
76	PNA	*Arachis hypogaea*	Natural	Vector	Galβ1-3GalNAc (T)
77	ACA	*Amaranthus caudatus*	Natural	Vector	Galβ1-3GalNAc (T)
78	HEA	*Hericium erinaceum*	Natural	JOM	Galβ1-3GalNAc (T)
79	ABA	*Agarics bisporus*	Natural	Vector	Galβ1-3GalNAc (T), GlcNAc
80	Jacalin	*Artocarpus integrifoli*	Natural	Seikagaku	Galβ1-3GalNAc (T), GalNAca (Tn)
81	MPA	*Maclura pomifera*	Natural	Seikagaku	Galβ1-3GalNAc (T), GalNAca (Tn)
82	HPA	*Helix pomatia*	Natural	Seikagaku	αGalNAc (A, Tn)
83	VVA	*Vicia villosa*	Natural	Vector	α,βGalNAc (A, Tn, LacDiNAc)
84	DBA	*Dolichos biflorus*	Natural	Vector	α,βGalNAc (A, Tn, LacDiNAc)
85	SBA	*Glycine max*	Natural	EY Lab.	α,βGalNAc (A, Tn, LacDiNAc)
86	rPPL	*Pleurocybella porrigen*	*E.coli*	AIST	α,βGalNAc (A, Tn, LacDiNAc)
87	rCNL	*Clitocybe nebularis*	*E.coli*	AIST	α,βGalNAc (A, Tn, LacDiNAc)
88	rXCL	*Xerocomus chrysenteron*	*E.coli*	AIST	Core1,3, agalacto N-glycan
89	VVA I	*Vicia villosa*	Natural	JOM	GalNAcβ1-3(4)Gal
90	WFA	*Wisteria floribunda*	Natural	Vector	Terminal GalNAc, LacDiNAc
91	rABA	*Agarics bisporus*	*E.coli*	AIST	Galβ1-3GalNAc (T), GlcNAc
92	rDiscoidin I	*Dictyostelium Discodeu*	*E.coli*	AIST	Gal
93	DBAIII	*Dioscorea batatas*	Natural	JOM	Maltose
94	rMalectin	*Homo sapiens*	*E.coli*	AIST	Glcα1-2Glc
95	CSA	*Oncorhynchus keta*	Natural	JOM	Rhamnose, Galα1-4Gal
96	FLAG-EW29Ch-E20K	*Lumbricus terrestris*	*E.coli*	AIST	6-sulfo-Gal

[1]Abbreviations: Gal (D-galactose), GalNAc (N-acetyl-galactosamine), GlcNAc (N-acetyl-glucosamine), Fuc (L-fucose), Glc (D-glucose), Sia (Sialic acid), LacNAc (N-acetyl-lactosamine).

[2]Specificity data was obtained by frontal affinity chromatography and glycoconjugate microarray.

[3]Abbreviations: JOM (J-OIL MILLS, INC), Vector (VECTOR LABORATORIES, INC), Seikagaku (SEIKAGAKU CORPORATION), EY (EY LABORATORIES, INC), AIST (National Institute of Advanced Industrial Science and Technology)

201B7(P48)

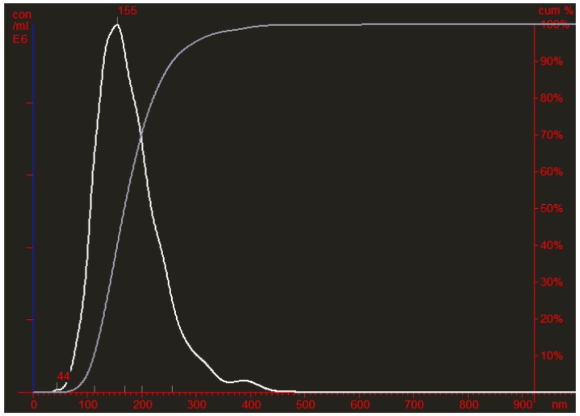

Particle Size / Concentration

Results	
Mean:	180 nm
Mode:	155 nm
SD:	60 nm
D10:	113 nm
D50:	169 nm
D90:	257 nm
User Lines:	0 nm, 0 nm
Concentration:	6.46 E8 particles/ml
Completed Tracks:	1128

ADSC#2118(P8)

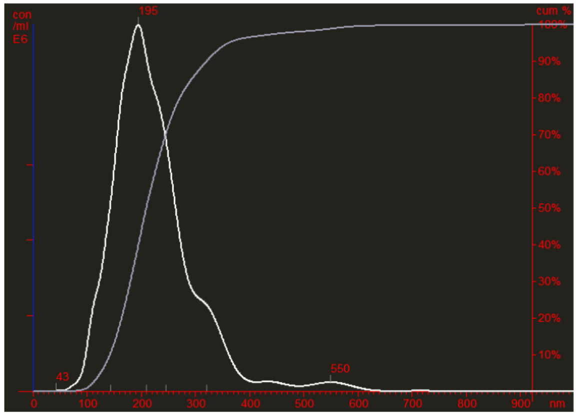

Particle Size / Concentration

Results	
Mean:	225 nm
Mode:	195 nm
SD:	81 nm
D10:	143 nm
D50:	210 nm
D90:	321 nm
User Lines:	0 nm, 0 nm
Concentration:	6.98 E8 particles/ml
Completed Tracks:	961

hFibs(P15)

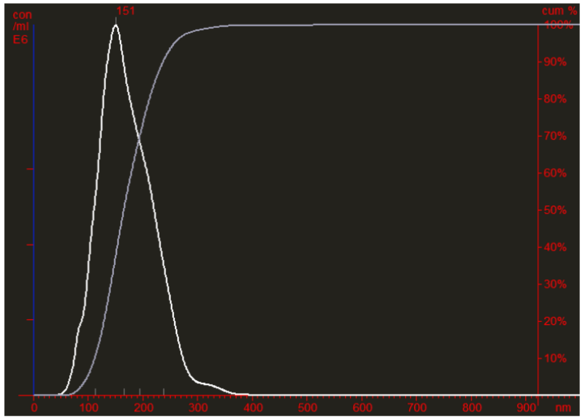

Particle Size / Concentration

Results	
Mean:	172 nm
Mode:	151 nm
SD:	48 nm
D10:	113 nm
D50:	166 nm
D90:	238 nm
User Lines:	0 nm, 0 nm
Concentration:	5.60 E8 particles/ml
Completed Tracks:	926

Fig. S1. Analysis of the particle sizes of EVs analyzed by Nanosight LM10 system.

73	rGal3C	*Homo sapiens*	*E.coli*	AIST	LacNAc, polylactosamine
74	rLSLN	*Laetiporus sulphureus*	*E.coli*	AIST	LacNAc, polylactosamine
75	rCGL3	*Coprinopsis cinerea*	*E.coli*	AIST	LacDiNAc
76	PNA	*Arachis hypogaea*	Natural	Vector	Galβ1-3GalNAc (T)
77	ACA	*Amaranthus caudatus*	Natural	Vector	Galβ1-3GalNAc (T)
78	HEA	*Hericium erinaceum*	Natural	JOM	Galβ1-3GalNAc (T)
79	ABA	*Agarics bisporus*	Natural	Vector	Galβ1-3GalNAc (T), GlcNAc
80	Jacalin	*Artocarpus integrifoli*	Natural	Seikagaku	Galβ1-3GalNAc (T), GalNAca (Tn)
81	MPA	*Maclura pomifera*	Natural	Seikagaku	Galβ1-3GalNAc (T), GalNAca (Tn)
82	HPA	*Helix pomatia*	Natural	Seikagaku	αGalNAc (A, Tn)
83	VVA	*Vicia villosa*	Natural	Vector	α,βGalNAc (A, Tn, LacDiNAc)
84	DBA	*Dolichos biflorus*	Natural	Vector	α,βGalNAc (A, Tn, LacDiNAc)
85	SBA	*Glycine max*	Natural	EY Lab.	α,βGalNAc (A, Tn, LacDiNAc)
86	rPPL	*Pleurocybella porrigen*	*E.coli*	AIST	α,βGalNAc (A, Tn, LacDiNAc)
87	rCNL	*Clitocybe nebularis*	*E.coli*	AIST	α,βGalNAc (A, Tn, LacDiNAc)
88	rXCL	*Xerocomus chrysenteron*	*E.coli*	AIST	Core1,3, agalacto N-glycan
89	VVA I	*Vicia villosa*	Natural	JOM	GalNAcβ1-3(4)Gal
90	WFA	*Wisteria floribunda*	Natural	Vector	Terminal GalNAc, LacDiNAc
91	rABA	*Agarics bisporus*	*E.coli*	AIST	Galβ1-3GalNAc (T), GlcNAc
92	rDiscoidin I	*Dictyostelium Discodeu*	*E.coli*	AIST	Gal
93	DBAIII	*Dioscorea batatas*	Natural	JOM	Maltose
94	rMalectin	*Homo sapiens*	*E.coli*	AIST	Glcα1-2Glc
95	CSA	*Oncorhynchus keta*	Natural	JOM	Rhamnose, Galα1-4Gal
96	FLAG-EW29Ch-E20K	*Lumbricus terrestris*	*E.coli*	AIST	6-sulfo-Gal

[1]Abbreviations: Gal (D-galactose), GalNAc (N-acetyl-galactosamine), GlcNAc (N-acetyl-glucosamine), Fuc (L-fucose), Glc (D-glucose), Sia (Sialic acid), LacNAc (N-acetyl-lactosamine).

[2]Specificity data was obtained by frontal affinity chromatography and glycoconjugate microarray.

[3]Abbreviations: JOM (J-OIL MILLS, INC), Vector (VECTOR LABORATORIES, INC), Seikagaku (SEIKAGAKU CORPORATION), EY (EY LABORATORIES, INC), AIST (National Institute of Advanced Industrial Science and Technology)

Fig. S2

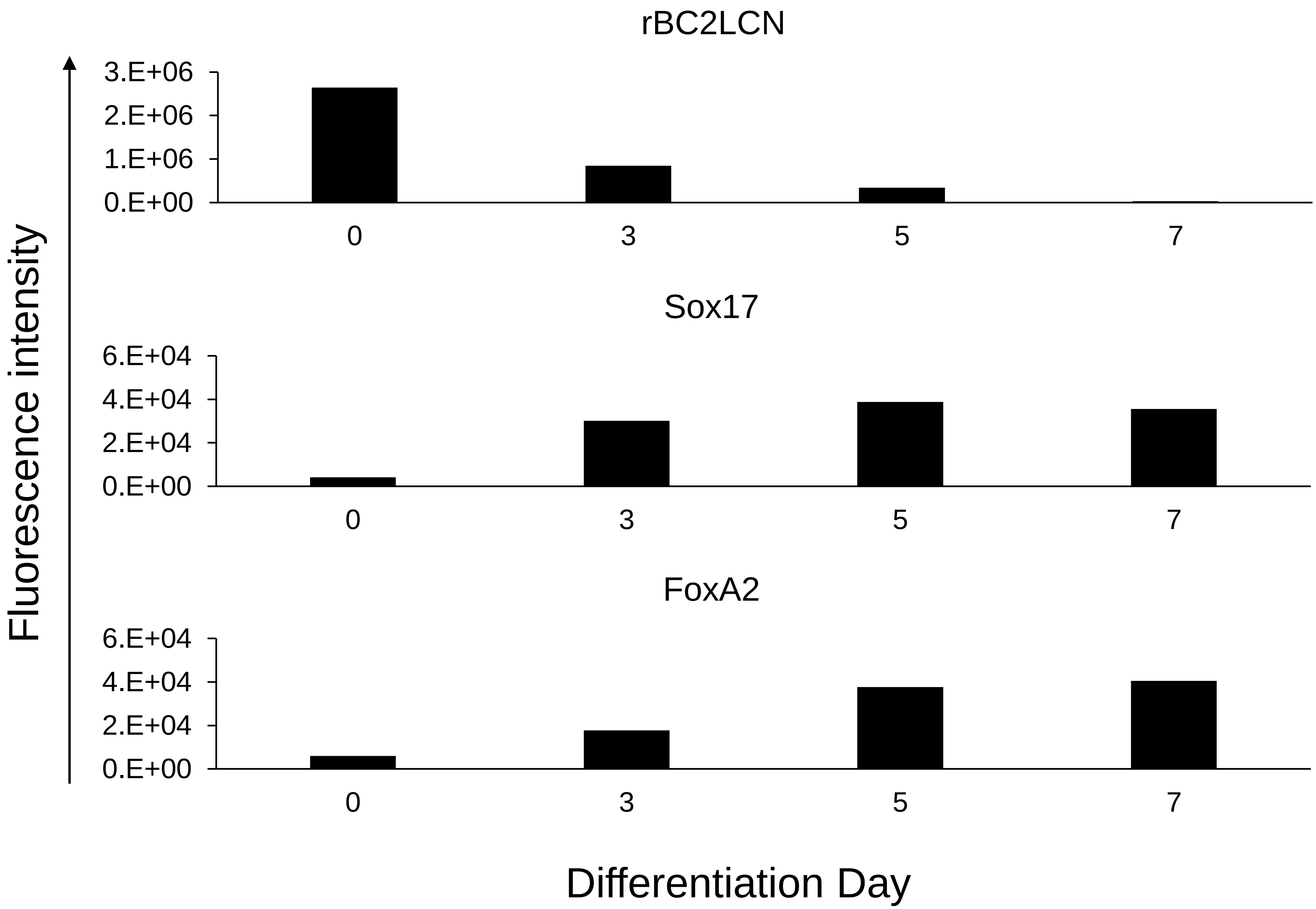

Fig. S2. Expression of rBC2LCN ligands and endoderm markers such as Sox17 and FoxA2 by flow cytometry. Data are shown as the mean fluorescence intensity.
